# Faecal microbiota changes associated with the moult fast in chinstrap and gentoo penguins

**DOI:** 10.1371/journal.pone.0216565

**Published:** 2019-05-08

**Authors:** Won Young Lee, Hyunjun Cho, Mincheol Kim, Binu Mani Tripathi, Jin-Woo Jung, Hosung Chung, Jeong-Hoon Kim

**Affiliations:** Division of Polar Life Sciences, Korea Polar Research Institute, Incheon, Republic of Korea; University of Oregon, UNITED STATES

## Abstract

In many seabirds, individuals abstain from eating during the moult period. Penguins have an intense moult that lasts for weeks, during which they are confined to land. Despite the importance for survival, it is still unclear how the faecal microbiota of Antarctic penguins changes in response to the moult fast. Here, we investigated the faecal microbiota of chinstrap (*Pygoscelis antarcticus*) and gentoo penguins (*Pygoscelis papua*) on King George Island, Antarctica. The bacterial community compositions during the feeding and moulting stages were compared for both species using bacterial 16S rRNA gene amplicon on an Illumina MiSeq platform. Our results showed that the moult fast altered the bacterial community structures in both penguin species. Interestingly, the bacterial community composition shifted in the same direction in response to the moult fast but formed two distinct clusters that were specific to each penguin species. A significant increase in bacterial diversity was observed in gentoo penguins, whereas no such change was observed for chinstrap penguins. By analysing the contribution of the ecological processes that determine bacterial community assembly, we observed that processes regulating community turnover were considerably different between the feeding and moulting stages for each penguin. At the phylum level, the relative abundances of *Fusobacteria*, *Firmicutes* and *Proteobacteria* were dominant in chinstrap penguins, and no significant changes were detected in these phyla between the feeding and moulting periods. Our results suggest that moult fast-induced changes in the faecal microbiota occur in both species.

## Introduction

Fasting behaviour is commonly observed in animals. For instance, many mammals hibernate during the harsh winter when there is no available food supply, whilst seabirds have an intensive moult-fast period on land to replace their feathers entirely. Especially for moulting birds, large amounts of energy are required to produce new feathers and to maintain essential physiological functions. Thus, the moult fast is a stressful period for individuals due to the energetic demands [1, 2].

Penguins are diving seabirds that also endure an intense moult that is confined to the land [3]. Due to the reduced thermal insulation during the moult, penguins must stay on land with no foraging in the sea. Thus, individuals utilize a large proportion of their body stores during the moult fast [4–6]. In Antarctica, because of its cold and harsh environment, penguins are predicted to be under more stress during the moult fast with respect to thermoregulation and feather synthesis. Recently, researchers have begun to investigate the gut microorganisms during the moult in penguins. Dewar et al. [7] examined the faecal bacterial community composition of penguins in temperate and sub-Antarctic regions during the moult fast including the little penguin, *Eudyptula minor*, in Australia and that of the king penguin, *Aptenodytes patagonicu*s, in South Georgia, respectively. They observed that the community composition of the gastrointestinal microbiota was altered in both penguin species during fasting.

However, little is known regarding how ecological processes govern the microbial community in the penguin gut ecosystem. The results of a few studies have indicated how ecological processes govern the microbial community in the gut ecosystem, and deterministic and stochastic processes were crucial in determining the composition of microbial communities [8, 9]. Moreover, recent findings suggest that some microbial patterns can also be explained by the same ecological mechanisms present in plants and animals [10–12]. In previous studies, the assembly of ecological communities were shown to be governed by four major processes (selection, dispersal, drift and mutation/speciation) [13, 14]. When a serious ecological disturbance occurs, the relative importance of these processes shift significantly within a specific ecosystem [12, 15]. The gut ecosystem of penguins during the moulting period presents unique features that make it especially attractive for addressing questions regarding the assembly of microbial communities. Both penguin species investigated in this study are unable to feed during the moulting period, and their food supply is limited. Because the lack of nutrition will eventually constrain the microbial community composition, the moulting event will affect the gut microbes by altering ecological processes for the microbial community in both penguin species.

In this study, we investigated how the faecal microbiota of Antarctic penguins changes during the moult-fast period. We assayed two species, the chinstrap penguin, *Pygoscelis antarcticus*, and the gentoo penguin, *Pygoscelis papua*, which are sympatric breeders in the Antarctic Peninsula. The two penguin species share similar life histories. In our study area, both penguins are highly dependent on Antarctic krill, *Euphausia superba*, and during the chick-rearing period approximately 99% of prey is Antarctic krill [16]). It was previously reported that different bacterial community compositions are present among four penguin species from different subfamilies (king, gentoo, macaroni and little penguin [17]), and the authors suggested that phylogeny and diet could be responsible for these differences. However, it remains unknown whether closely related penguins with similar dietary habits share faecal microbiomes.

This study focused on two closely related penguin species of the genus *Pygoscelis*, which offer an ideal system to assess how the composition of faecal bacterial communities varies between closely related penguin species with similar dietary preferences during the moult-fast period. In addition, we investigated the effects of moulting on the major ecological processes that govern microbial communities in the gut of two closely related penguin species.

## Materials and methods

### Ethical statement

This research was conducted under the approval from the Korean Ministry of Foreign Affairs and Trade and according to the current laws of the Republic of Korea (‘Act on Antarctic Activities and Protection of Antarctic Environment’, certificate paper number: ILAD-4029 [2012.11.19]). The certificate paper permits conducting research in Antarctic Specially Protected Area No. 171 (Narębski Point) and includes consideration and approval of handling chinstrap and gentoo penguins for faecal sampling. Local ethics committee from Ministry of Environment of the Republic of Korea reviewed and approved this work.

All applicable international, national, and/or institutional guidelines for the care and use of animals were followed.

### Study site and sample collection

This study was conducted in a chinstrap and gentoo penguin colony at Narębski Point (Antarctic Specially Protected Area, No. 171; 62°13'40''S-62°14'23''S, 58°45'25''W-58°47'00''W) in Barton Peninsula on King George Island, Antarctica in January and February 2013. At this site, there are approximately 3,000 and 2,500 breeding pairs of chinstrap and gentoo penguins, respectively. For each penguin species, we collected faeces samples on two separate occasions, the chick-guarding period during feeding (before the moult-fast) and after the chick-rearing period, which occurs in the middle of the moult period during an approximately 2–3 week fasting period [18, 19]. Among the breeding adults, seven chinstrap and seven gentoo penguins were randomly sampled during feeding (10^th^ January of 2013) and six gentoo and five chinstrap penguins during moulting (12^th^ February), with 25 samples collected in total.

For faecal sampling, each penguin was restrained in a cotton bag with its flippers and legs held tightly. Sterilized swab were gently inserted approximately 1–2 inches into the cloaca. After sampling the faeces, the tip of the swab was stored into a 1.5 ml tube, and the tubes were kept at -20°C.

### DNA extraction and PCR amplification

Faecal DNA was extracted using a MO BIO PowerSoil DNA Isolation kit (MoBio Laboratories, Carlsbad, CA, USA) following manufacturer’s instructions. The isolated DNA was stored at -80°C in a deep freezer until PCR was performed. The bacterial community structure in each faeces sample was assessed using an Illumina MiSeq sequencing platform targeting the V3-V4 region of the bacterial 16S rRNA gene, and the community compositions during feeding and during moulting were compared within and between the two penguin species. The V3-V4 region of the bacterial 16S rRNA gene was amplified using primers the Bakt_341F (5’-CCTAGGGGNGGCWGCAG-3’) and Bakt_805R (5’-GACTACHVGGGTATCTAATCC-3’) [20], along with sequencing primer and adapter sequences for MiSeq sequencing.

Polymerase chain reaction (PCR) was performed in a 25 µl reaction volumes and contained 2.5 μl of genomic DNA extract, 5 μl of each primer (1 μM), 12.5 μl of KAPA HiFi Hotstart ReadyMix (Kapa Biosystems Ltd., London, UK). The PCR thermocycling conditions used were as follows: 95°C for 3 min for an initial denaturation followed by 25 cycles of 95°C for 30 s, 55°C for 30 s, and 72°C for 30 s, with a final extension of 72°C for 5 min. The resultant amplicons were purified using AMPure XP beads (Beckman Coulter, Indianapolis, IN, USA). The purified amplicons were indexed using Illumina Nextera XT index kit (Illumina, San Diego, CA, USA) and then used for paired-end sequencing (2 × 300 nt) with an Illumina MiSeq system (Illumina, San Diego, CA, USA).

### Data analysis and sequence processing

The paired-end 16S rRNA gene sequences were assembled using the assembler PANDAseq [21]. Merged reads were processed using mothur [22] following the mothur MiSeq SOP (http://www.mothur.org/wiki/MiSeq_SOP). The sequences were aligned against the SILVA-aligned reference sequences and were then further filtered to remove gaps. The sequences were de-noised using the ‘pre.cluster’ command in mothur implementation of a pseudo-single linkage pre-clustering algorithm from Huse and colleagues [23]. Putative chimeric sequences were detected and removed via the Chimera Uchime algorithm contained within mothur [24] in de novo mode, which first splits sequences into groups and then checks each sequence within a group using the more abundant groups as references. Chimera-free sequences were taxonomically classified down to the genus level based on EzBioCloud 16S database [25]. The quality-filtered sequences were clustered into operational taxonomic units (OTUs) using an OptiClust algorithm with a threshold of 97% sequence similarity. Non-bacterial reads assigned to Archaea and Eukaryotes were excluded, and all of the singleton OTUs were removed from all datasets prior to further analysis. The MiSeq sequence data used in this study are deposited in the MG-RAST server [26] under project ID 84568 (https://www.mg-rast.org/linkin.cgi?project=mgp84568).

### Predictive functional profiling by PICRUSt

The potential functional profiles of faecal microbiotas were predicted with PICRUSt v.1.1.0 [27], which uses an ancestral state reconstruction algorithm to predict metagenomic functional profiles from 16S rRNA gene sequence data and a reference genome database. An OTU table that was produced using a closed-reference OTU picking process was used as an input table. The taxonomic information for each OTU was determined using the Greengenes database v13.5 [28] and then was used to show the relative distribution of shared OTUs. The OTU table was first normalized by 16S rRNA gene copy number predictions and then the metagenomes were predicted and summarised at the level 2 KO (KEGG Orthology) category.

### Statistical analysis

All of the samples were standardized by random subsampling to 13,415 sequences per sample using the “sub.sample” command in mothur to correct for differences in number of reads between samples. All bacterial sequences were rarefied to the lowest number of reads generated from any sample. Bray-Curtis dissimilarities among all sample pairs were calculated on a Hellinger-transformed OTU abundance matrix. Non-metric multidimensional scaling (NMDS) was used to visualize the moulting effects on the bacterial community composition of each penguin group using the ‘metaMDS’ function in the vegan R package [29]. A centroid in the ordination space was calculated and ellipses illustrating standard deviations of the community structures within each stage were drawn using the ‘ordiellipse’ function in R. The program STAMP (version 2.1.3) was used to test for significant differences in the functional profiles between different treatment groups [30]. Welch’s t-test [31] was performed to compare functional profiles between two different treatment groups along with effect size measures and confidence intervals set to 95%.

Permutational multivariate analysis of variance (PERMANOVA), a semiparametric multivariate test, was used to assess differences in bacterial community structure between the chinstrap and gentoo penguins during feeding and moulting using PRIMER 6 & PERMANOVA+ [32]. Species (chinstrap and gentoo) and stage (feeding and moulting) were included as fixed factors, and *p*-values were obtained using 999 permutations. Welch’s two sample t-tests were used to compare the feeding and moulting groups with respect to bacterial diversity and relative abundance for the two penguin species.

### Phylogenetic analysis

To estimate the relative influence of stochastic and deterministic processes, we employed a phylogenetic null modelling approach to calculate β-nearest taxon index (βNTI). βNTI is the difference between the observed β-mean nearest taxon distance (βMNTD) and the mean of the null distribution of βMNTD normalized by its standard deviation, and calculated using ‘Picante’ R package and custom scripts as described earlier [33, 34]. βNTI values greater than +2 and less than -2 indicate dominance of variable (more than expected phylogenetic turnover) and homogenous selection (less than expected phylogenetic turnover), respectively; and values between +2 and -2 indicate dominance of stochastic processes. We employed a second null model in combination with βNTI referred as Bray-Curtis-based Raup-Crick (RC_bray_) as described by Stegen et al. [33] to further partitioned stochastic processes into homogenizing dispersal, dispersal limitation and undominated process (drift was referred to as “undominated” processes in Stegen et al. [35]). The percentage of pairwise comparisons with |βNTI| < 2 but RC_bray_ > +0.95 or < −0.95 indicate the relative influences of dispersal limitation or homogenizing dispersal, respectively. On the other hand, the fraction of pairwise comparisons with |βNTI| < −2 and |RC_bray_| < 0.95 indicate that the shift in community composition is resulted due to random drift.

## Results

A total of 1,516,737 high quality 16S rRNA gene sequences that were assigned to 19,990 operational taxonomic units (OTUs) at > 97% similarity level from 27 samples after the removal of low quality and chimeric sequences. The bacterial read numbers and OTU richness in each sample are shown in the supplementary material (S1 Table).

**Fig 1:**
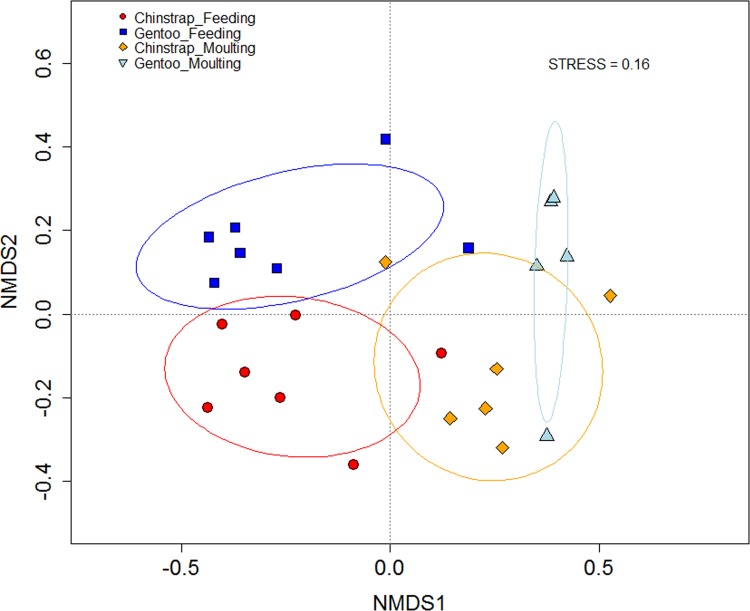
NMDS plot of faecal bacterial community structures in chinstrap penguin and gentoo penguins during feeding and moulting. The bacterial structures formed distinct clusters specific to each penguin species but shifted in the same direction (chinstrap penguin during feeding (n = 7) and moulting (n = 6) and gentoo penguins during feeding (n = 7) and moulting (n = 5)); PERMANOVA, df = 1, mean square = 6069.3, Pseudo F = 1.92, p = 0.006 for interaction of Species × Moult stage). Samples were grouped by ellipses enclosing all points in each group using the ‘ordiellipse’ function in the vegan R package.

Faecal bacterial communities were distinctly structured by both factors (penguin species and moult stage), and although the bacterial community structures shifted in the same direction along the first NMDS axis in both penguin species, two distinct clusters specific to each penguin species formed, suggesting a potential interaction effect of species × moult (Table 1, PERMANOVA, p = 0.006 for interaction of Species × Moult stage; see NMDS plot in Fig 1).

**Table 1:** Statistical results of PERMANOVA to estimate the effects of species (chinstrap and gentoo) and moulting stage (during feeding and moulting).

Source	df	Mean square	Pseudo-F	P-value
Species (chinstrap and gentoo)	1	8047.4	2.55	0.002
Stage (during feeding and moulting)	1	11830	3.74	0.001
Species × Stage	1	6069.3	1.92	0.006
Residual	23	3161.9	-	-
Total	26	-	-	-

**Fig 2:**
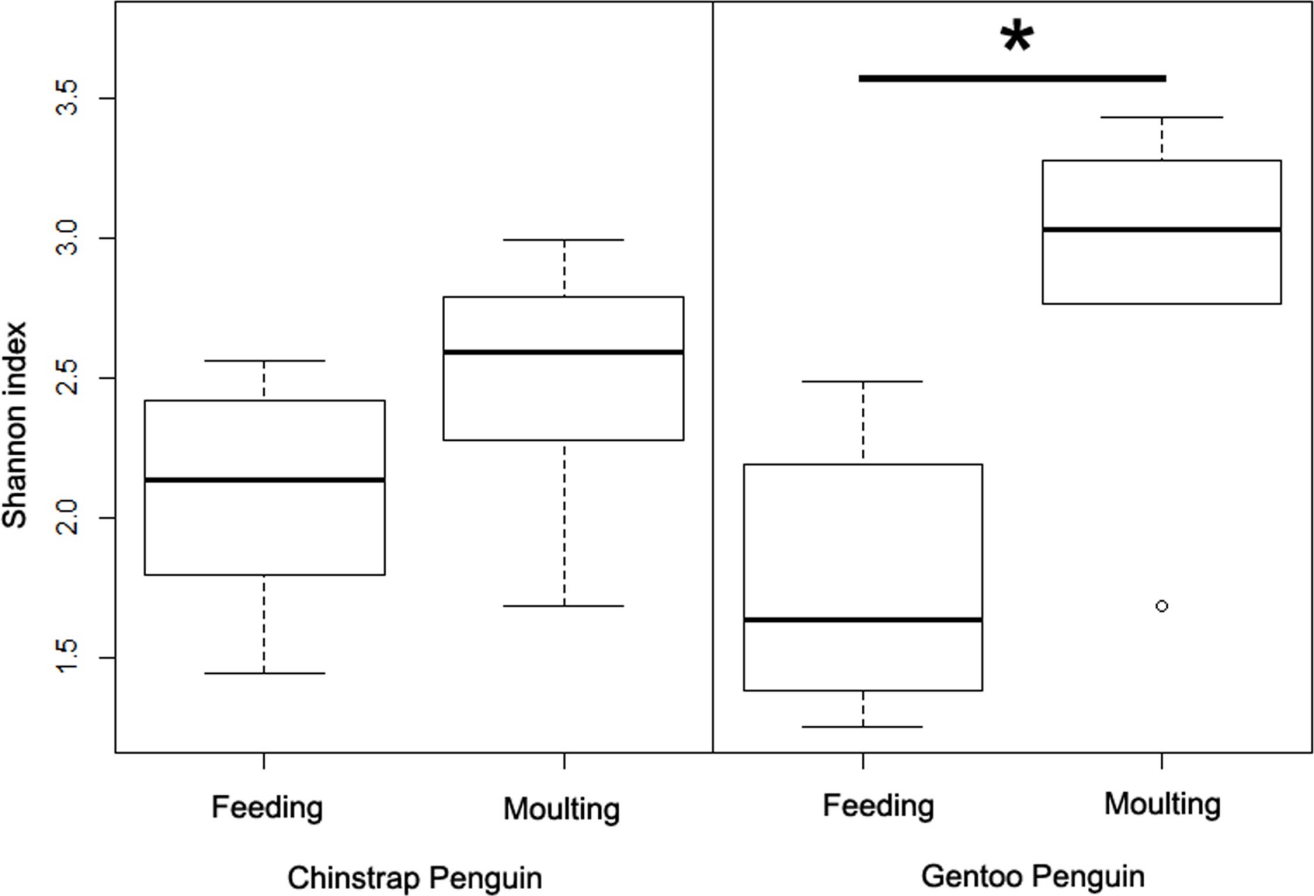
Bacterial diversity (Shannon index) in chinstrap (left) and gentoo penguins (right) during the feeding and moulting stages. The lower quartiles represent 25% of data and the upper quartiles represent 75% of data. The asterisk indicates the significant difference between the feeding and moulting gentoo penguin groups (p < 0.05).

Shannon’s diversity index, which measures the number of species and takes into account species evenness, indicated that bacterial diversity did not change during the moult in chinstrap penguins (Welch’s two sample t-test, t = -1.61, df = 10.36, p = 0.14; Fig 2) but increased in gentoo penguins (Welch’s two sample t-test, t = -2.89, df = 6.91, p = 0.02; Fig 2).

**Fig 3:**
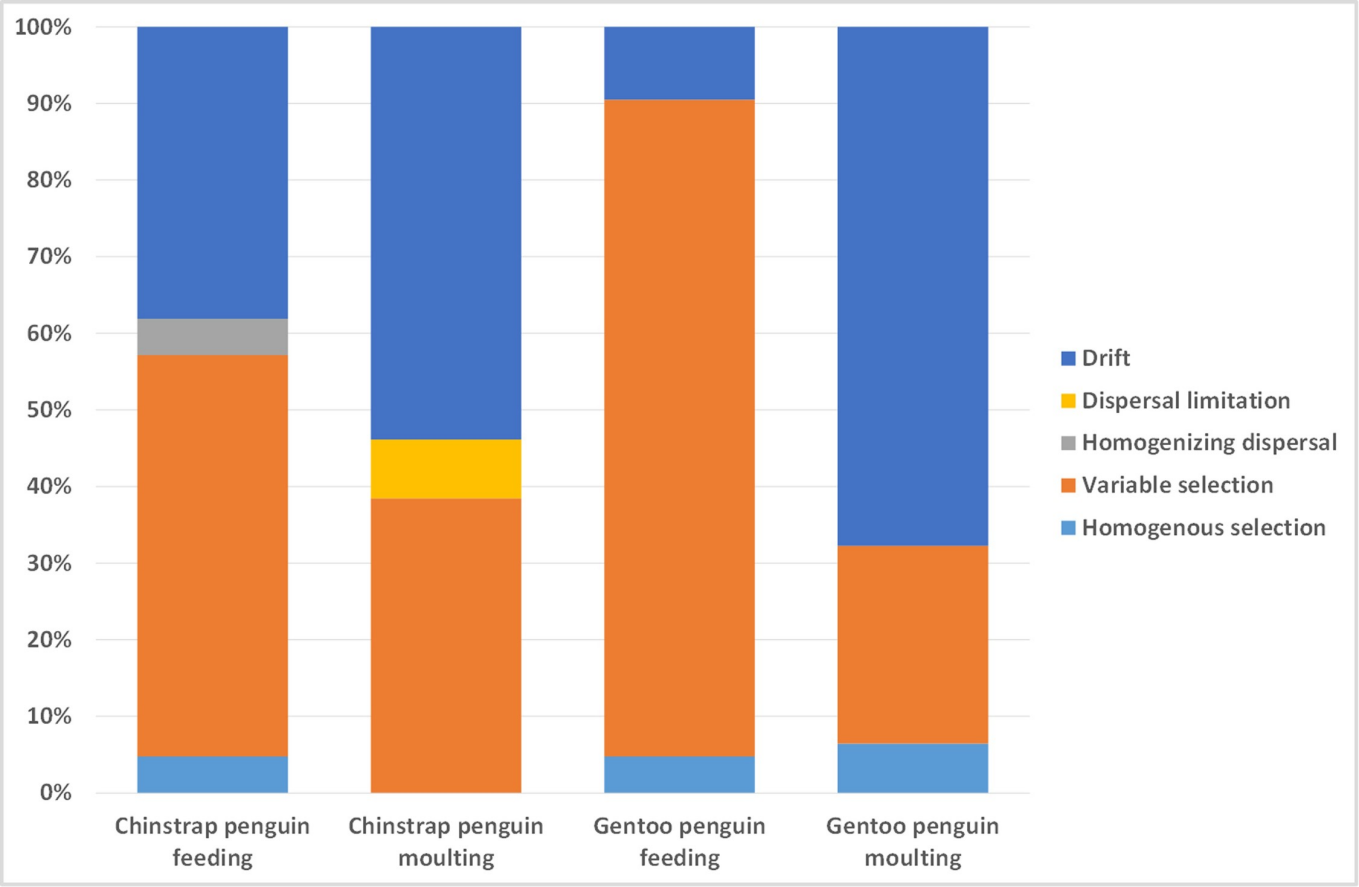
Summary of the contributions of the ecological processes that determine bacterial community assembly of the faecal microbiota in chinstrap and gentoo penguins. The percent of turnover in bacterial community assembly was governed by various deterministic (homogeneous and variable selection) and stochastic processes (dispersal limitation, homogenizing dispersal and drift).

By further quantifying the relative contributions of major ecological processes that structure faecal microbiota, we observed that the processes regulating community turnover differ considerably between the feeding and moulting stages for each penguin species (Fig 3). Generally, the contributions of specific ecological processes showed similar patterns for both the feeding and moulting stages in each species, but the magnitude of these contributions differed between the two species. Variable selection was more pronounced in the feeding stage (chinstrap penguins: 52.38%; gentoo penguins: 85.71%), whereas drift played an important role in the assembly of the faecal microbiota at moulting stage in both penguin species (chinstrap: 46.67%; gentoo: 50%).

**Fig 4:**
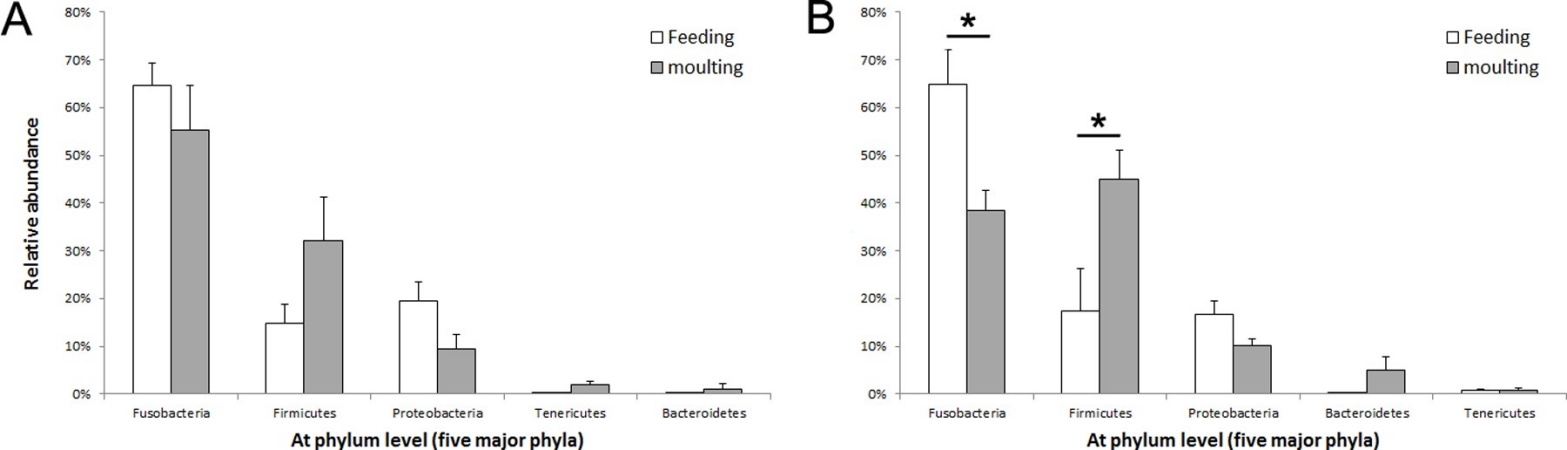
**Relative abundances of dominant bacterial phyla in the chinstrap (a) and gentoo penguin (b) faecal bacterial communities during feeding (empty bar) and moulting (grey bar).** The relative abundances of major phyla (upper) and genera (bottom) accounted for more than 99% of total number of OTUs (chinstrap penguin, feeding (n = 7) and moulting (n = 6); gentoo penguin, feeding (n = 7) and moulting (n = 5); t-tests, asterisks indicate significance with a p-value of less than 0.05).

**Fig 5:**
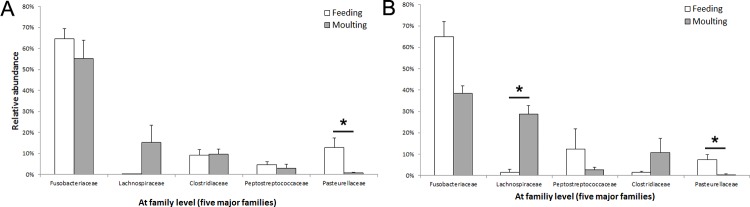
**Relative abundances of dominant bacterial families in the chinstrap (a) and gentoo penguin (b) faecal bacterial communities during feeding (empty bar) and moulting (grey bar).** The relative abundances of major phyla (upper) and genera (bottom) accounted for more than 99% of total number of OTUs (chinstrap penguin, feeding (n = 7) and moulting (n = 6); gentoo penguin, feeding (n = 7) and moulting (n = 5); t-tests, asterisks mean significance of p-value less than 0.05).

The relative abundances of major bacterial phyla, which represented more than 99% of all sequences, are presented in Fig 4. In chinstrap penguins, *Fusobacteria* (64.69 and 55.28%), *Firmicutes* (14.84 and 32.17%), and *Proteobacteria* (19.42 and 9.32%) were observed to be abundant during feeding and moulting, respectively. No significant differences were observed in the abundances of these phyla between the two stages (t-test, all p > 0.05). In the gentoo penguins, *Fusobacteria* (64.92 and 38.53%), *Firmicutes* (17.31 and 45.09%), *Proteobacteria* (16.69 and 10.21%), and *Bacteroidetes* (0.21 and 4.95%) were dominant in samples from both the feeding and moulting stages, respectively. Among these phyla, the relative abundance of *Firmicutes* during the moulting stage compared to the feeding stage was significantly increased (t-test, t = 0.52, p = 0.04), while that of *Fusobacteria* was decreased (t-test, t = 0.51, p = 0.02) (Fig 4B). The relative abundances of the dominant families observed are presented in Fig 5. In the chinstrap penguins, *Fusobacteriaceae* (64.68 and 55.26%), *Lachnospiraceae* (0.24 and 15.19%), *Clostridiaceae* (9.23 and 9.68%), *Peptostreptococcaceae* (4.56 and 3.08%), and *Pasteurellaceae* (12.90 and 0.69%) were detected in samples from the feeding and moulting stages, respectively. Among these, only *Pasteurellaceae* decreased significantly after moulting (t-test, t = 0.51, p = 0.03). In the gentoo penguins, *Fusobacteriaceae* (64.92 and 38.51%), *Lachnospiraceae* (1.53 and 28.73%), *Peptostreptococcaceae* (12.48 and 2.68%), *Clostridiaceae* (1.40 and 10.77%), and *Pasteurellaceae* (7.51 and 0.37%) were detected during feeding and moulting, respectively. Among these taxa, the abundance of *Lachnospiraceae* increased significantly after moulting (t-test, t = 0.50, p < 0.001), whereas that of *Pasteurellaceae* decreased significantly after moulting (t-test, t = 0.51, p = 0.02). The taxonomic profiles at the genus levels of chinstrap and gentoo penguins, which are belonging to the major families, were provided in the supplementary material (S1 Fig).

The PICRUSt results revealed the predicted functional pathway changes during penguin feeding and moulting (S2 Fig and S1 Table). In the chinstrap penguins, the relative abundances of the membrane transport and metabolism functional pathways decreased, whereas that of transport and catabolism, folding, sorting and degradation, glycan biosynthesis and metabolism and cellular processes and signalling increased. In the gentoo penguins, the relative abundances of many metabolic pathways decreased, including, cell mobility, amino acid metabolism, biosynthesis of other secondary metabolites, carbohydrate metabolism and lipid metabolism. In contrast, the relative abundances of other pathways increased, including genetic information processing, translation, nucleotide metabolism, replication and repair, folding, and sorting and degradation.

## Discussion

In our study, the bacterial faecal community structures between chinstrap and gentoo penguins were observed to be significantly different between the feeding and moulting stages. The results of previous studies of the faecal bacteria of penguins also indicated the interspecific differences (king, gentoo, macaroni and little penguins [17]) at different habitats. In this study, the chinstrap and gentoo penguins studied are close sympatric breeders in the same genus (*Pygoscelis*) and share very similar food sources (this population exclusively feeds on Antarctic krill, *E*. *superba* [16]). Despite the close phylogenetic relationship and similar diet, the two penguin species exhibited different faecal bacterial communities during the feeding and moulting stages (Fig 1).

By evaluating OTU richness, we detected a significant increase in bacterial diversity in gentoo but not chinstrap penguins during the moulting stage. This result may have occurred from the abundances of the dominant bacterial taxa having decreased due to resource depletion during feeding, allowing other minor taxa to have increased in abundance during the moulting stage. Increased microbial diversity resulting from fasting has been reported in various taxa (hamsters [36]; pythons [37]; tilapia, toads, and mice [38]), but it has been shown that this is not a universal effect across taxa [38]. Fasting may also decrease microbial density resulting from decreased levels of food in the gut (hamsters [36]; reindeers [39]) and reduced intestinal size (blackcaps, *Sylvia atricapilla* [40] and domestic broilers [41]). Thus limited nutrient availability may promote increased competition among taxa and result in increased bacterial diversity.

By quantifying the relative contributions of ecological processes that structure faecal microbiota in both investigated penguin species, we observed that processes regulating community turnover differed considerably by moulting period. Generally, variable selection and drift are two major ecological processes that structure faecal microbiota in these closely related Antarctic penguin species. The selection process was pronounced during the feeding period (before moulting) in both penguin species, while drift play an important role in the assembly of the gut microbiota during the moulting period. One of the possible explanations of these findings could be that nutrient poor conditions in gut of moulting penguins reduced abundance of gut microbiota and that communities with smaller population sizes were more susceptible to drift [42, 43]. However, the causes of variation in the magnitude of the contribution of ecological processes that determine community assembly of the gut microbiota in both penguins are unclear. Because both penguin species share similar diets, this variation may be due to the different roles of bacterial taxa that dominated in each penguin species.

The relative abundances of dominant bacterial phyla or families showed distinct patterns in each penguin species. At the phylum level, no significant changes were observed in the faecal microbiota of the chinstrap penguins between the two sampling periods. However, the relative abundance of *Firmicutes* increased and that of *Proteobacteria* decreased in gentoo penguins after moulting. This result was not observed in previous studies of king and little penguins [7]. At the family level, *Pasteurellaceae*, which belong to the phylum *Proteobacteria*, decreased during the moult in both species (chinstrap penguins, 12.90 to 0.69%; gentoo penguins, 7.51 to 0.37%). *Pasteurellaceae* is a large group of gram-negative chemoorganotrophic, facultatively anaerobic, and fermentative bacteria [44]. During the moult, the growth of chemoorganotrophic bacteria could be negatively affected by the limited food supply. *Fusobacteriaceae* and *Clostridiaceae*, two primary families in both species, are widely known pathogens in vertebrates and were both reported to be abundant in the guts of black and turkey vultures [45] and alligators [46]. Both vultures and alligators feed on decaying prey. Interestingly, genes encoding tissue-degrading enzymes were detected in the gut metagenome of the turkey vulture [45]. Furthermore, by outcompeting other bacteria, these bacterial families may contribute to the carrion digestion while tolerating bacterial toxins [45, 47]. We speculate that these bacteria may play roles in digesting remained food in the gut under restricted food conditions but the mechanisms may differ from that of the scavenging birds.

In contrast, the abundance of the family *Lachnospiraceae*, which belong to the phylum *Firmicutes*, increased during the moult for both species, but was only significant for the gentoo penguins (chinstrap penguins, 0.24 to 15.19%; gentoo penguins, 1.53 to 28.73%). *Lachnospiraceae* is a family of bacteria in the order *Clostridiales* that is commonly present in chicken caeca [48] and produce butyric acid in human [49]. The results of a recent study showed that dietary sodium butyrate improves intestinal development in broilers and functions by modulating the microbial community [50]. Moreover, it has been increasing identified that a higher level of *Lachnospiraceae* stabilizes the intestinal environment by retarding the accumulation of lactate [51, 52]. The growth of *Lachnospiraceae* bacteria family during the moulting period could be positively affected by the limitation of food supply. For both penguin species, the phyla *Fusobacteria*, *Firmicutes*, *Proteobacteria*, *Bacteroidetes* and *Tenericutes* were dominant (more than 99% of sequences) in the feeding stage samples. These results agree with those obtained for the faecal bacterial compositions of king, gentoo, macaroni and little penguins (*Bacteroidetes*, *Firmicutes*, *Proteobacteria*, and *Fusobacteria* [17]; S2 Table) and the stomach bacterial compositions of chinstrap and Adelie penguins (Fusobacteria, Firmicutes, *Tenericutes* and *Proteobacteria* [53]; S2 Table). The major bacterial phyla associated with penguins have been reported to be distinct from those of other birds. While Fusobacteria is commonly detected as a major taxon in penguin studies during breeding season (S2 Table), including in this study, the shared major bacterial phyla in wild birds are Firmicutes, *Proteobacteria*, *Bacteroidetes* and *Actinobacteria* [54]. It may be that the distinctive dominance of *Fusobacteria* in penguins has an important role in penguin biology. Interestingly, members of the phylum *Fusobacteria* are known butyrate-producing bacteria, and their ecological importance was discussed in previous studies for fatty acid metabolism during the moult fast [7] and for chick survival at early growing stages [55]. Thus, *Fusobacteria* is also regarded to be an important bacterial taxon that affect gentoo and chinstrap penguins during the feeding and moulting stages.

What are the mechanisms that cause the observed changes in penguin faecal bacterial communities during the moult fast? The initial step is the lack of a food supply by the host animals, which may provide different environmental conditions for bacteria. During the moulting period, the penguins excrete greenish liquid faeces, the colour of which is due to bile [56]. This environmental change could decrease the dominant bacterial taxa during feeding and increase those that prefer the environmental conditions of the gut during moulting. In this study, we suggest that fasting behaviour involves changes faecal microbiota. However, the hormonal changes and immune responses in penguins during this period have yet to be elucidated, and these data may provide important information on why such bacterial changes occur.

The PICRUSt results showed the functional pathway changes that occurred during the two sample periods, and both species exhibited decreases in metabolic pathways (membrane transport and metabolism in chinstrap penguins; amino acid metabolism, biosynthesis of other secondary metabolites, carbohydrate metabolism and lipid metabolism in gentoo penguins) and increases in biosynthetic replication and repair pathways (folding, sorting and degradation in chinstrap penguins; replication and repair, folding, sorting and degradation in gentoo penguins) during the moult. This result may indicate that faecal bacteria were under low-nutrient conditions with no external energy supply and the presence of bile acids, which influence the pH in the gut. The results of a previous metagenomics study showed that starvation may induce the enrichment of microbial genes related to antibiotic activity and host genes related to the immune system [57]. However, it should be noted that these changes may not reflect changes in gene expression, due to the limitation of the computational predictions and the expected modelling using a reference genome database.

In summary, we observed that the moult-fast altered the faecal bacterial community structure of two Antarctic penguin species. In response to the moult-fast period, the bacterial community composition shifted in the same direction but formed two distinct clusters specific to each species of penguin. However, increased bacterial diversity was only detected in the gentoo penguins, suggesting that moult-fast periods in penguin species can change the faecal microbiota and that specific bacterial phyla or families may play roles in determining the bacterial community structure in both the pre-moult and post-moult periods. Moreover, although the contribution of the ecological processes that determine gut microbiota community assembly in both penguin species showed similar patterns, the magnitude of these contributions were different between the two species. This result might be due to the specific roles of dominant bacterial taxa in each species.

In future studies, metagenomic analyses will be necessary to reveal the functional roles of microorganisms in moulting penguins. Penguins also have a fasting period during incubation, and male king penguins were observed to maintain a pH value at 6 and reduced gastric motility throughout the three-week incubation fasting period to preserve stomach contents [58]. Thus, it would be interesting to study how gut microbiota changes with other fasting periods.

## Supporting information

S1 Fig Taxonomic profiles at the genus levels of chinstrap and gentoo penguin faecal bacterial communities during feeding and moulting, belonging to the major families (A) *Fusobacteriaceae*, (B) *Lachnospiraceae*, (C) *Peptostreptococcaceae*, (D) *Clostridiaceae*, and (E) *Pasteurellaceae*.
(TIF)

S2 Fig Predicted functional pathway changes during feeding (green bar) and moulting (yellow bar) by PICRUSt in gentoo (A) and chinstrap (B) penguins.
(TIF)

S1 Table Summary of sample information and MiSeq amplicon sequencing results across 25 samples. (DOCX)

S2 Table Summary of previous studies on penguin gut microbiota. (DOCX)
