# Supplementary figures and images for: Faecal microbiota changes associated with the moult fast in chinstrap and gentoo penguins
Source: PLoS One. 2019 May 8;14(5):e0216565. doi: 10.1371/journal.pone.0216565 (PMC6505947; doi:10.1371/journal.pone.0216565)

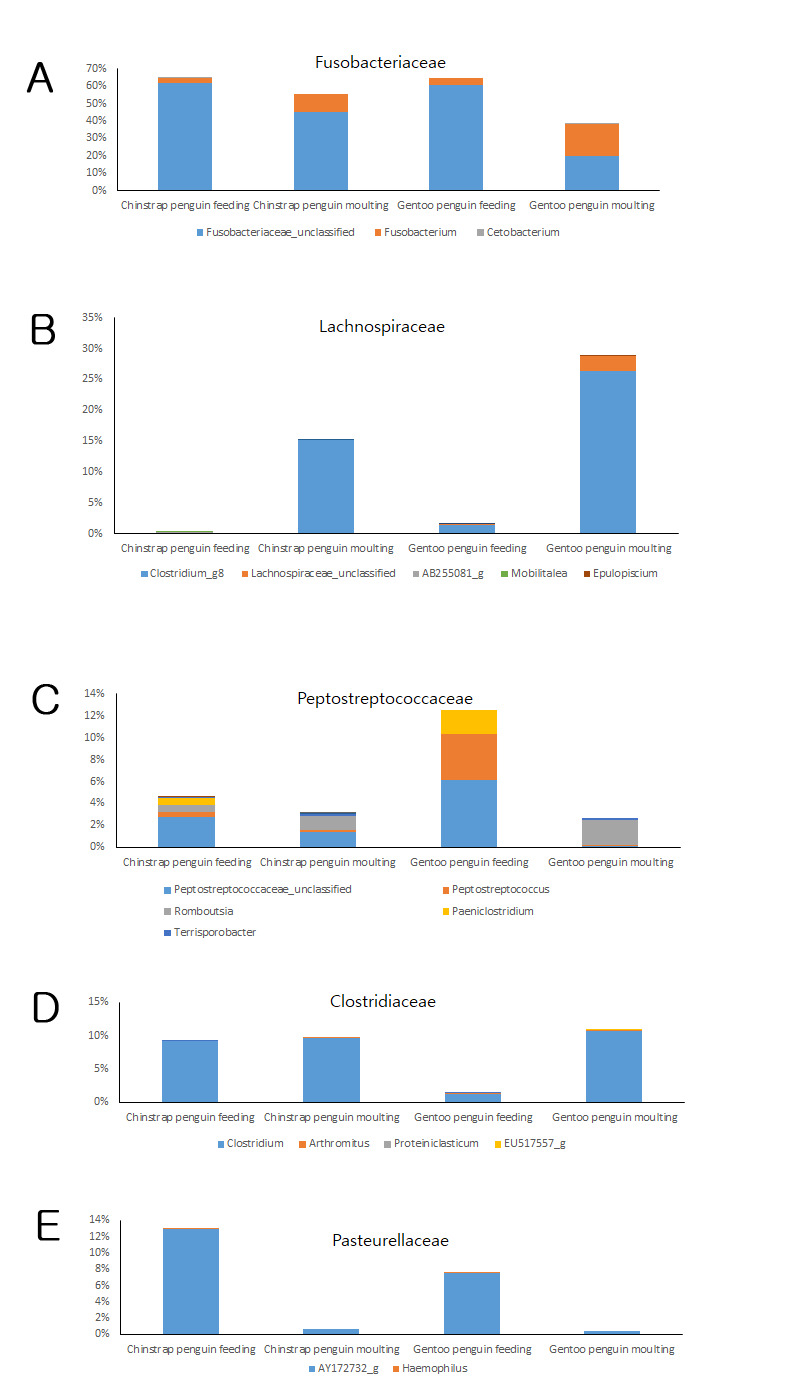

Supplement: S1 Fig — Taxonomic profiles at the genus levels of chinstrap and gentoo penguin faecal bacterial communities during feeding and moulting, belonging to the major families (A) Fusobacteriaceae, (B) Lachnospiraceae, (C) Peptostreptococcaceae, (D) Clostridiaceae, and (E) Pasteurellaceae. (TIF) [file pone.0216565.s001.tif]

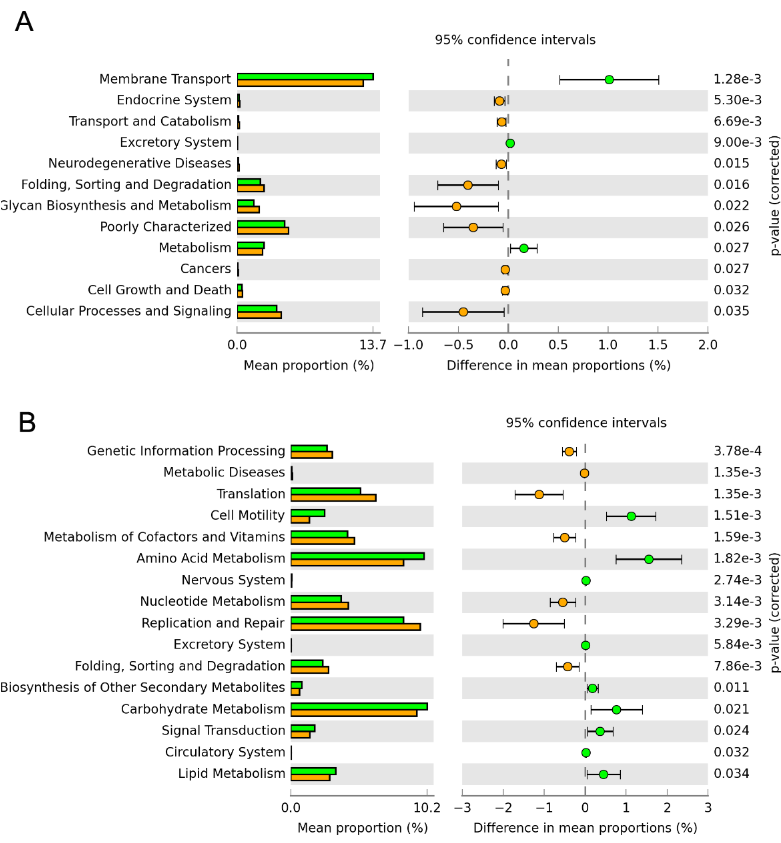

Supplement: S2 Fig — Predicted functional pathway changes during feeding (green bar) and moulting (yellow bar) by PICRUSt in gentoo (A) and chinstrap (B) penguins. (TIF) [file pone.0216565.s002.tif]
