# Supplementary material for: Faecal microbiota changes associated with the moult fast in chinstrap and gentoo penguins
Source: PLoS One. 2019 May 8;14(5):e0216565. doi: 10.1371/journal.pone.0216565 (PMC6505947; doi:10.1371/journal.pone.0216565)
Supplement: S1 Table — (DOCX) [file pone.0216565.s003.docx]

**Supplementary Table 1.** Summary of sample information and MiSeq amplicon sequencing results across 25 samples,

| **Sample name** | **Species** | **Stage** | **Bacterial reads** | **OTU richness*** |
| --- | --- | --- | --- | --- |
| CF1 | Chinstrap | Feeding | 99,320 | 612 |
| CF2 | Chinstrap | Feeding | 66,579 | 692 |
| CF3 | Chinstrap | Feeding | 42,695 | 759 |
| CF4 | Chinstrap | Feeding | 62,632 | 736 |
| CF5 | Chinstrap | Feeding | 61,455 | 546 |
| CF6 | Chinstrap | Feeding | 38,766 | 591 |
| CF7 | Chinstrap | Feeding | 53,892 | 702 |
| CM1 | Chinstrap | Moulting | 64,888 | 483 |
| CM2 | Chinstrap | Moulting | 48,485 | 739 |
| CM3 | Chinstrap | Moulting | 45,722 | 806 |
| CM4 | Chinstrap | Moulting | 51,980 | 730 |
| CM5 | Chinstrap | Moulting | 62,537 | 706 |
| CM6 | Chinstrap | Moulting | 35,182 | 811 |
| GF1 | Gentoo | Feeding | 44,956 | 606 |
| GF2 | Gentoo | Feeding | 43,666 | 913 |
| GF3 | Gentoo | Feeding | 36,984 | 849 |
| GF4 | Gentoo | Feeding | 33,879 | 1102 |
| GF5 | Gentoo | Feeding | 53,532 | 825 |
| GF6 | Gentoo | Feeding | 44,280 | 826 |
| GF7 | Gentoo | Feeding | 43,207 | 682 |
| GM1 | Gentoo | Moulting | 87,098 | 429 |
| GM2 | Gentoo | Moulting | 52,140 | 930 |
| GM3 | Gentoo | Moulting | 30,567 | 712 |
| GM4 | Gentoo | Moulting | 45,174 | 1008 |
| GM5 | Gentoo | Moulting | 50,285 | 904 |
|  |  |  |  |  |
| * OTU richness was calculated for 30,567 randomly generated subsets per sample. | | | | |
