# Supplementary material for: Faecal microbiota changes associated with the moult fast in chinstrap and gentoo penguins
Source: PLoS One. 2019 May 8;14(5):e0216565. doi: 10.1371/journal.pone.0216565 (PMC6505947; doi:10.1371/journal.pone.0216565)
Supplement: S2 Table — (DOCX) [file pone.0216565.s004.docx]

**Supplementary Table 2.** Summary of previous studies on penguin gut microbiota.

| Penguin species | Reference | Major phylum (%) |
| --- | --- | --- |
| Little penguin | Dewar et al. 2013 | Proteobacteria (30), Firmicutes (24), Bacteroidetes (22), Planctomyces (11), Actinobacteria(6) |
|  | Dewar et al. 2014 | Firmicutes (from 44 to 29), Bacteroidetes (from 11 to 20) (from early to late moult) |
| King penguin | Dewar et al. 2013 | Firmicutes (47), Bacteroidetes (17), Proteobacteria (4), Fusobacteria (3) |
|  | Dewar et al. 2014 | Fusobacteria (from 1.73 to 33.6) proteobacteria (from 35.7 to 17.2) Bacteroidetes (from 19.5 to 11) (from early moult to late moult) |
| Macaroni penguin | Dewar et al. 2013 | Firmicutes (43), Proteobacteria (30), Bacteroidetes (18), Actinobacteria (3), Spirochaeta (1) |
| Gentoo penguin | Dewar et al. 2013 | Actinobacteria (55), Proteobacteria (18), Firmicutes (18), Bacteroidetes (7), Actinobacteria (1) |
| Adélie penguin | Banks et al. 2009 | Firmicutes (39), Actinobacteria (30, ) Others (17), Bacteroidetes (10), Fusobacteria (5) |
| Chinstrap penguin | Barbosa et al. 2016 | Firmicutes (60), Bacteroidetes (17.5), Proteobacteria (11), Fusobacteria (9), Actinobacteria (3.6), Tenericutes (1.7) |
